# FDA-Approved Antibacterials and Echinocandins

**DOI:** 10.3390/antibiotics14020166

**Published:** 2025-02-07

**Authors:** Othman Al Musaimi

**Affiliations:** 1 School of Pharmacy, Newcastle University, Newcastle upon Tyne NE1 7RU, UK; othman.almusaimi@newcastle.ac.uk; 2 Department of Chemical Engineering, Imperial College London, London SW7 2AZ, UK

**Keywords:** peptides, pharmaceuticals, FDA, antimicrobial, echinocandin, bacteria, fungus

## Abstract

Since 1955, a total of 12 peptide-based drugs with antimicrobial or antifungal properties have received approval from the Food and Drug Administration (FDA). Peptides present a promising opportunity to address serious infections that may be challenging to manage through other means. Peptides exhibit the capability to leverage various mechanisms, and in some cases, multiple mechanisms are employed for this purpose. Despite the initial approval dating back to 1955, the FDA recently approved an echinocandin peptide just last year. The ongoing approvals underscore the significance of peptides in addressing ongoing medical challenges. Approximately 22 peptide therapeutics with an antibacterial and antifungal spectrum are currently undergoing various phases of clinical trials, showing promising results. In this review, antimicrobial and antifungal peptides are analyzed in terms of their chemical structure, indication, mode of action, and development journey, concluding with their arrival in the pharmaceutical market.

## 1. Introduction

The extraordinary growth in the global pharmaceutical industry has now spread to include peptides [1]. Thanks to their tolerable safety profile, biocompatibility, biodegradability, and specificity, they are considered an appealing class of drugs. They have been incorporated in various medical fields, including cardiology [2], oncology [3], wound treatment, and others [4,5]. Therapeutic peptides are well suited to address existing unmet medical challenges, making them excellent complements and sometimes more preferable alternatives to small molecules and very large biologics [6]. With their medium size (500–5000 Da) [7], peptides possess a substantial binding footprint for interacting with therapeutic targets, often outperforming small molecules. This allows them to bind to cell surface receptors and act through agonism/antagonism mechanisms [8]. Additionally, peptides can exert their biological effects through direct translocation into cells, surpassing the capabilities of large biologics [9,10]. Peptides are utilized as targeted therapy to deliver a range of payloads to their therapeutic targets, encompassing small molecules, peptides, and large proteins [3,7,11,12]. A well-known example of a commonly administered peptide is insulin [13]. At present, there are around 120 peptide drugs on the global market, and research into new peptide therapeutics continues at a steady pace, with more than 150 peptides in clinical development and another 400–600 peptides undergoing preclinical studies [14]. These new medicines require pharmaceutical companies to adopt new synthetic strategies to deliver these peptide therapeutics more effectively and in a green and sustainable fashion wherever possible [15].

The UK pharmaceutical industry, valued at USD 53 billion annually, accounts for 2.6% of the global pharmaceutical market. Peptides, monoclonal antibodies, and oligonucleotides constitute the three largest and fastest-growing classes of new biotherapeutic products under development. Projections indicate that the peptide therapeutic market will expand from USD 38 billion to USD 106 billion between 2023 and 2033, with a compound annual growth rate (CAGR) of 10.8% [16]. Over the last five years, the FDA has approved 23 peptides for a range of applications, including therapeutic, diagnostic, and, in some instances, theranostic uses, representing almost half of the approvals seen in the previous decade [1,17]. This indicates a growing prominence of peptides, with expectations to secure a significant share by 2030, potentially surpassing the threshold of 60 approvals.

Antimicrobial peptides (AMPs) typically comprise 10–50 amino acid residues, and are found in a variety of organisms, including plants (e.g., *Brassica species*) [18], insects (e.g., fruit fly *Drosophila melanogaster*) [19], amphibians (e.g., African clawed frog *Xenopus laevis*) [20], and mammals (e.g., humans *Homo sapiens*) [21]. They exhibit a wide range of effectiveness against various microbes including bacteria, viruses, and fungi [22,23,24]. The existing antibiotic regimen has been excessively utilized and is now confronted with the challenge of microbial resistance [22]. The surge in multidrug-resistant (MDR) bacteria has resulted in increased infections and higher mortality rates, and poses a significant threat to global health [25,26]. Hence, the advancement of various strategies to counter infections is steadily underway. Antimicrobial peptides (AMPs) have undergone extensive research and demonstrate promising potential as antibiotics [27,28]. The primary advantage of AMPs lies in their distinct mode of action, specifically their ability to interact with microbial membranes and distinguish between host and pathogenic cells [29]. Their antimicrobial properties and selectivity enable them to effectively target and eliminate bacteria within mixed cultures. A key reason for their antimicrobial efficacy and safety is that mammalian cells typically feature neutral zwitterionic phospholipid-containing bilayer membranes, which lack electrostatic attraction to AMPs. Additionally, the higher cholesterol content in mammalian cells provides another basis for differentiation from bacterial cells [30]. Lipids and proteins form the phospholipid bilayer, the core structure of cell membranes. Phosphatidylcholine (PC) and phosphatidylethanolamine (PE) are typically uncharged, while phosphatidylserine (PS), cardiolipin (CL), and phosphatidylglycerol (PG) are negatively charged. PS, CL, and PG are common in bacterial pathogens but rare in mammalian membranes, where PC and PE dominate. Eukaryotic membranes contain sterols like cholesterol (mammals) and ergosterol (fungi), which are scarce in prokaryotes. Gram-negative bacteria have lipopolysaccharide (LPS), and Gram-positive bacteria have lipoteichoic acid, both adding negative charges. In fungi, negative charges come from phosphomannan and components like phosphatidylinositol (PI), PS, and diphosphatidylglycerol (DPG) [31,32].

Resistance mechanisms observed against traditional antibiotics like *β*-lactams, aminoglycosides, quinolones, and fluoroquinolones are ineffective against AMPs. Consequently, AMPs hold promise for treating infections caused by antibiotic-resistant microbes [33,34]. Moreover, the mode of action of AMPs on microbial membranes suggests that microbial resistance to AMPs is challenging to develop. Microbes would potentially need to undergo significant restructuring of their cell membranes to acquire resistance. Given that such reorganization would require changes to enzymes across various pathways, the likelihood of rapid adaptation leading to resistance is low.

This review aims to provide insights into FDA-approved analogs of antimicrobial peptides and echinocandins. The analysis will encompass their chemical structures, indications, mode of action, administration routes, development journey, and adverse effects.

## 2. Antimicrobial Peptides (AMPs)

The FDA has granted approval for seven antimicrobial peptides, categorized as common peptide structures, glycopeptides (containing glycosylated cyclic or polycyclic peptides), lipopeptides (containing a lipid (fat) component), and lipoglycopeptides (combining features of both lipids (fats) and glycopeptides) (Table 1).

Glycopeptides consist of a peptide and a sugar unit, with varied classes determined by substituents and the type of residues at positions 1 and 3 of the heptapeptide [35]. These peptides are used in the treatment of severe infections caused by methicillin-resistant *Staphylococcus aureus* (MRSA) or *Enterococcus* bacteria resistant to *β*-lactams and other antibiotics. Notably, they operate independently of penicillin-binding proteins (PBPs), enabling them to overcome the PBP mutations responsible for MRSA’s resistance to *β*-lactams.

**Figure 1:**
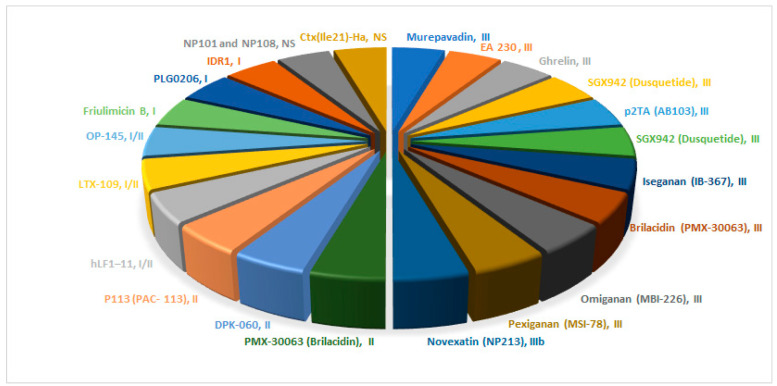
AMPs undergoing clinical trials and their corresponding phase.

Several antimicrobial agents are currently undergoing different phases of clinical trials (Figure 1) [36]. Cresti and colleagues have provided a concise overview of AMPs progressing through various clinical phases [37].

**Figure 2:**
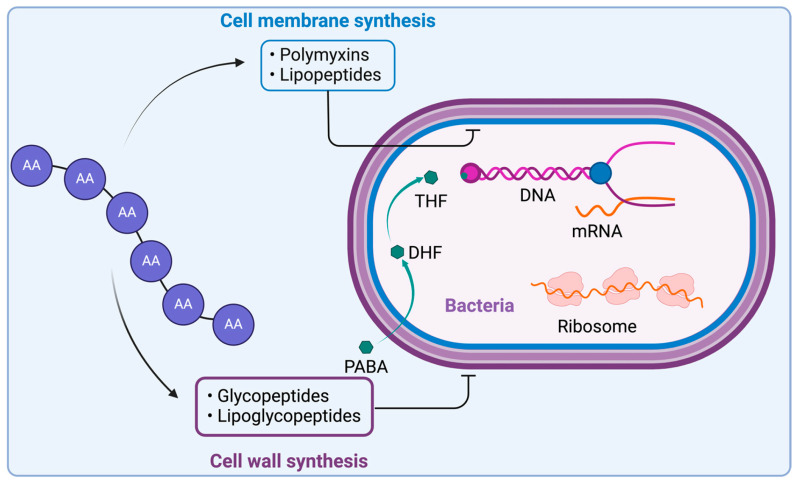
General mechanism of action of AMPs. Lipopeptides disrupt the bacterial cell membrane through electrostatic interactions between their positively charged amino acid residues and the negatively charged membrane components. Glycopeptides and lipoglycopeptides inhibit cell wall synthesis by binding to the tetrapeptide motif, preventing its involvement in peptidoglycan synthesis. Created with www.biorender.com (accessed on 19 January 2025).

AMPs can be categorized as membrane-acting or non-membrane-acting. Membrane-acting AMPs primarily target anionic microbial membranes, leading to disruptions in the membrane structure, whereas non-membrane-acting peptides facilitate membrane translocation without causing membrane destruction (Figure 2) [38]. The membrane-targeting mechanisms of AMPs can be explained using various models, such as the carpet model and the pore model. The pore model can be further categorized into the toroidal pore and barrel-stave models. AMPs enter cells through direct penetration or endocytosis. Once inside the cytoplasm, they identify and interact with specific targets. Based on these targets, AMPs can be categorized into distinct groups [39]. Enninful et al. has extensively detailed the major mechanisms of AMP–membrane interactions [40].

Glycopeptides exert their inhibitory effect on cell wall biosynthesis by binding to tetrapeptide chains, hindering their linkage by the PBP enzyme. Specifically, they bind to the D-Ala-D-Ala terminus of *N*-acetylmuramic acid (NAM) and *N*-acetylglucosamine (NAG) peptides, preventing their incorporation into peptidoglycan, a crucial component of the cell wall (Figure 2).

### 2.1. Gramicidin D (Neocidin)

**Figure 3:**
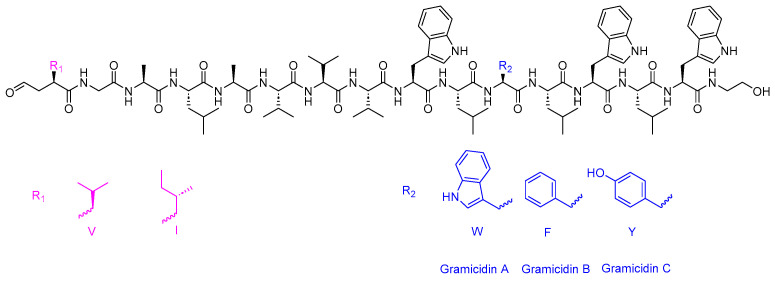
Chemical structure of gramicidin D.

Gramicidin D is a 15-mer linear acid peptide, and it is composed of a heterogeneous mixture of three pore-forming peptides, A (80%), B (5%), and C (15%) (Figure 3) [41,42]. Gramicidin D is used to treat infected surface wounds, as well as eye, nose, and throat infections.

Gramicidin D exhibits a robust binding affinity towards cell membranes, particularly targeting the membranes of Gram-positive bacteria. This interaction disrupts and permeabilizes the membrane, serving as a channel. Consequently, a cascade of detrimental effects ensues, including (i) depletion of intracellular solutes such as K+ and amino acids; (ii) dissipation of the trans-membrane potential; (iii) inhibition of respiration; (iv) reduction in ATP pools; and (v) interference with DNA, RNA, and protein synthesis, ultimately leading to cell death (refer to the colistin mechanism of action, Section 2.3).

In 1939, gramicidin was initially discovered in the soil bacterium Bacillus brevis [43]. In 1964, the sequence of gramicidin A was elucidated by Reinhard Sarges and Bernhard Witkop [44,45]. Gramicidin was approved by the FDA in 1955. Gramicidin D is not administered intravenously/systemically due to the risk of hemolysis, where significant intake may lead to the rupture of red blood cells. Therefore, it is typically used in the form of a lotion or ointment. Side effects may include redness, burning, stinging, or itching of the eye or ear, as well as blurred vision.

### 2.2. Vancomycin (Vancocin)

**Figure 4:**
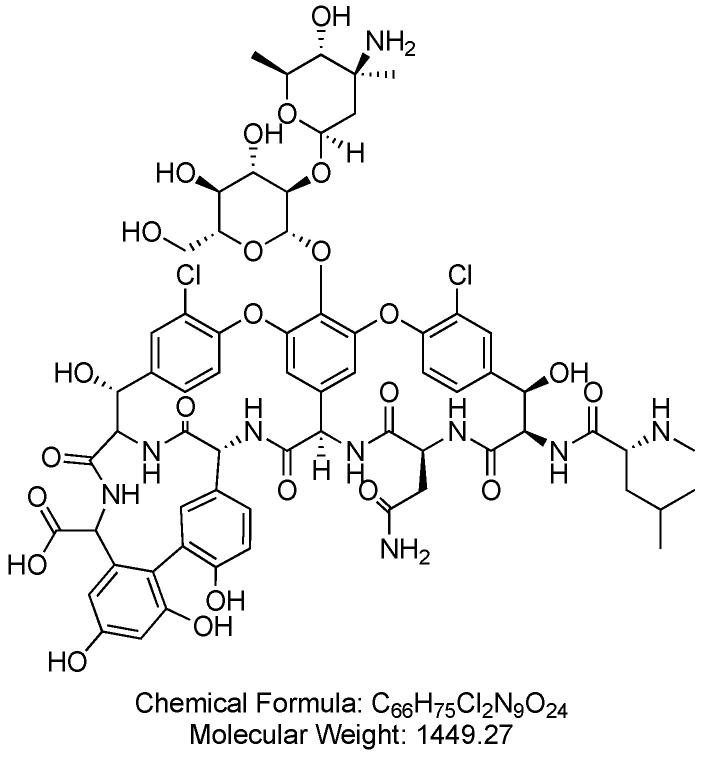
Chemical structure of vancomycin. CID. 14969.

Vancomycin is a tricyclic glycopeptide antibiotic originally derived from the organism *Streptococcus orientalis* (Figure 4). When administered intravenously, it is used to treat conditions such as septicemia, infective endocarditis, skin and skin structure infections, bone infections, and lower respiratory tract infections. When administered orally, it is used to treat clostridioides difficile-associated diarrhea and enterocolitis caused by *Staphylococcus aureus*, including strains that are methicillin-resistant [46].

**Figure 5:**
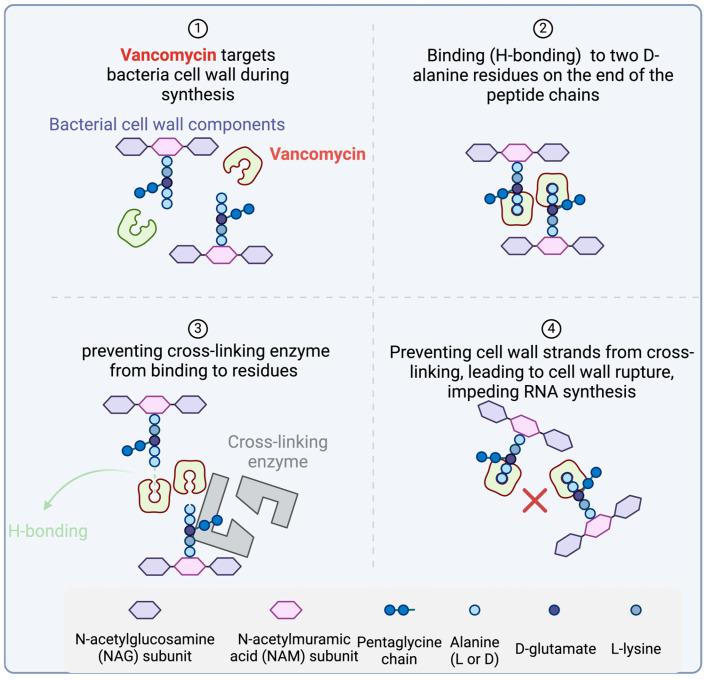
Vancomycin mechanism of action. Vancomycin binds to D-alanyl-D-alanine residues, blocking peptidoglycan synthesis in Gram-positive bacteria. This disrupts cell wall formation, alters membrane permeability, and inhibits RNA synthesis, leading to bacterial cell death. Created with www.biorender.com (accessed on 19 January 2025).

Vancomycin forms hydrogen bonds with the terminal D-alanyl-D-alanine moieties present in the NAM and NAG peptide subunits (Figure 5). This interaction hinders their integration into the peptidoglycan matrix, the primary structural element of Gram-positive cell walls. As a result, the antibiotic disrupts cell wall synthesis, causing changes in bacterial–cell–membrane permeability and impeding RNA synthesis [46].

Zhejiang Novus Pharmaceuticals developed vancomycin, and it received FDA approval in 1958 [46]. Vancomycin can be administered both orally and intravenously, with associated side effects such as acute kidney injury, hearing loss, neutropenia, anaphylaxis, and vancomycin infusion reaction. The predominant adverse reactions observed with oral administration include nausea, abdominal pain, and hypokalemia [46].

### 2.3. Colistin (Coly-Mycin M)

**Figure 6:**
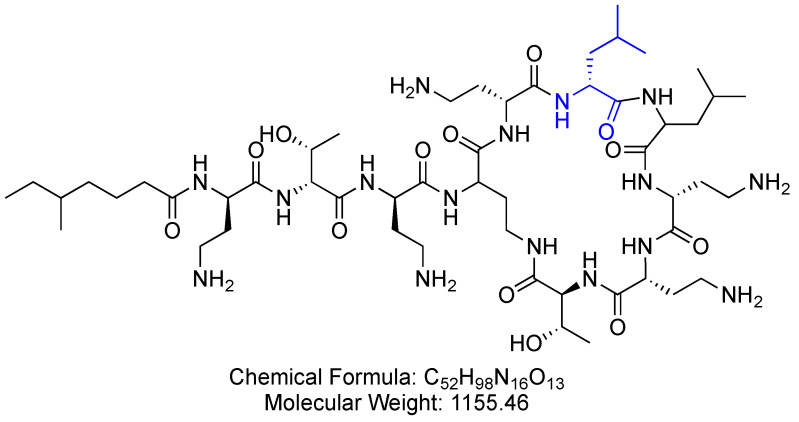
Chemical structure of colistin. Blue—in polymyxin b, a D-Phe residue replaces the D-Leu residue. CID. 5311054.

Colistin is a 10-mer lipopeptide, a polymyxin antibiotic. It comprises three parts, hydrophobic acyl tail, linear tripeptide, and hydrophilic heptapeptide (Figure 6) [47]. Colistin is used for the treatment of infections caused by MDR Gram-negative bacteria [48].

**Figure 7:**
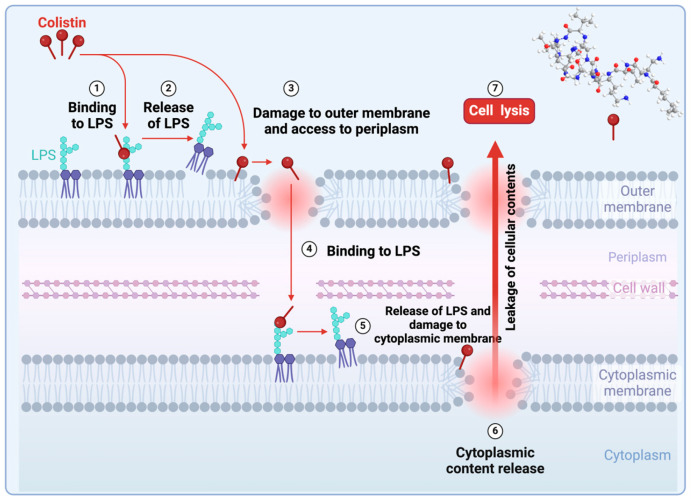
Colistin mechanism of action. Colistin binds LPS, displacing Mg^2+^ and Ca^2+^, disrupting membrane integrity, and causing cell leakage in Gram-negative bacteria. Created with www.biorender.com (accessed on 19 January 2025).

The mechanism entails the interaction between the cationic cyclic segment of colistin and the anionic lipopolysaccharide (LPS) molecules, resulting in the displacement of Mg^2+^ and Ca^2+^ ions from the outer cell membrane of Gram-negative bacteria. Consequently, this interaction induces permeability changes in the cell envelope, ultimately leading to the leakage of cellular contents (Figure 7) [49].

Colistin was developed by JHP Pharmaceuticals and received FDA approval in 1959 [49]. Colistin is administered intravenously and may entail side effects such as gastrointestinal upset, tingling of extremities and tongue, slurred speech, dizziness, vertigo, paresthesia, generalized itching, urticaria, rash, and fever [48].

Polymyxin B and colistin both belong to the polymyxin class, and they are used for similar indications. Polymyxin B received FDA approval in 1964 [50]. Polymyxin B can be administered via several routes including intramuscular, intravenous drip, intrathecal, or ophthalmic use. However, it is associated with various adverse effects such as nephrotoxicity (kidney damage), neurotoxicity (nerve damage), drug fever, urticarial rash (hives), and pain at injection sites. Thrombophlebitis (inflammation of veins) can also occur at intravenous injection sites [50].

### 2.4. Daptomycin (Cubicin)

**Figure 8:**
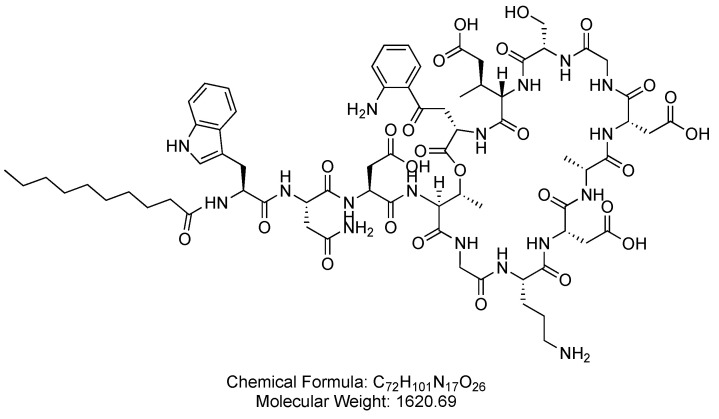
Chemical structure of daptomycin. CID. 21585658.

Daptomycin is a cyclic lipopeptide, derived from the fermentation of *Streptomyces roseosporus* (Figure 8). Daptomycin is used for the treatment of skin and skin structure infections caused by Gram-positive bacteria, *S. aureus* bacteremia, and right-sided *S. aureus* endocarditis.

**Figure 9:**
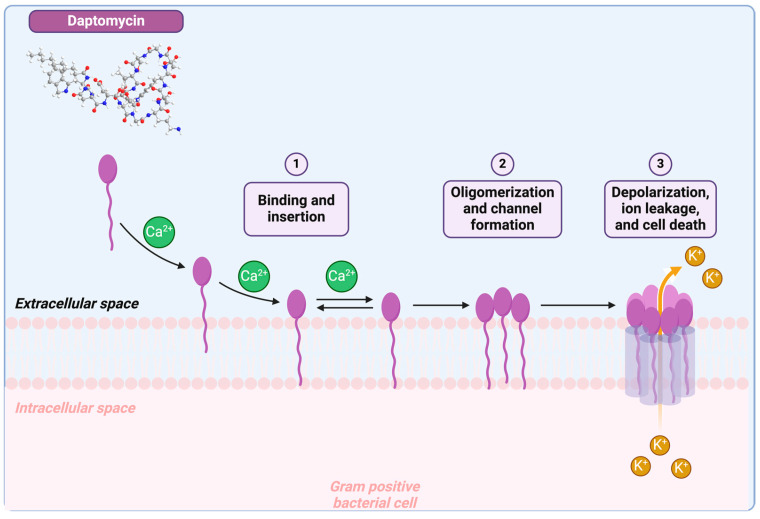
Daptomycin mechanism of action. Daptomycin binds bacterial membranes, causing depolarization through ion leakage, which inhibits protein, DNA, and RNA synthesis, leading to cell death. Created with www.biorender.com (accessed on 19 January 2025).

Daptomycin operates through a distinct mechanism of action by binding to bacterial membranes, inducing rapid depolarization of their potential through ion leakage [51]. This action leads to the inhibition of protein, DNA, and RNA synthesis, ultimately resulting in bacterial death (Figure 9) [52,53]. Huang conducted a more in-depth exploration to elucidate its distinctive mechanism [54]. Daptomycin forms a Dap₂Ca₃PG₂ complex in the membrane, acting as a transient ionophore. It binds and releases ions at the membrane boundary, facilitating ion transport from high to low concentrations. Mobility is key to its function, with only small dimeric complexes likely acting as ionophores. Over time, daptomycin aggregates grow, eventually exiting the membrane in a lipid-extracting effect [55], supporting its transient ionophore role [54].

Daptomycin was first discovered by in 1980s by researchers at Eli Lilly and company in soil samples from Mount Ararat in Turkey [53]. Subsequently, it was developed by Cubist Pharmaceuticals and obtained FDA approval in 2003 [56]. Daptomycin is administered intravenously and may elicit adverse effects such as anemia, anxiety, asthenia, constipation, diarrhea, dizziness, fever, flatulence, gastrointestinal discomfort, headache, hypertension, hypotension, increased risk of infection, insomnia, nausea, pain, skin reactions, and vomiting [52].

### 2.5. Telavancin (Vibativ)

**Figure 10:**
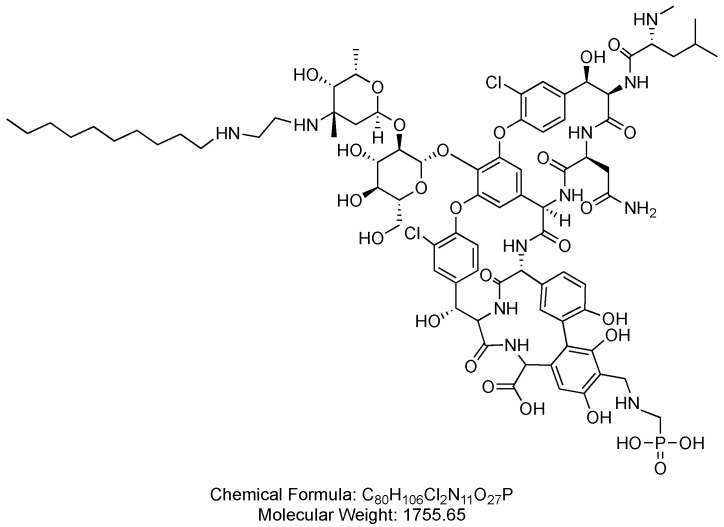
Chemical structure of telavancin. CID. 3081362.

Telavancin is a 7-mer lipoglycopeptide, derived from vancomycin. It consists of a lipophilic side chain (decylaminoethyl) attached to an amino sugar, and a hydrophilic moiety (phosphonomethyl aminomethyl) at the 4′-position of amino acid 7, anchored to a vancosamine sugar (Figure 10). Telavancin is used in the treatment of various infections in adult patients, including complicated skin and skin structure infections (CSSSIs), as well as hospital-acquired and ventilator-associated bacterial pneumonia (HABP/VABP) caused by *Staphylococcus aureus* [57].

Telavancin binds to late-stage peptidoglycan precursors, including lipid II, thereby preventing the polymerization of NAM and NAG, as well as the cross-linking of peptidoglycan by binding to D-Ala-D-Ala. This dual action ultimately leads to the inhibition of cell wall synthesis. Additionally, telavancin binds to the cell membrane, disrupting its function as a barrier [57,58].

Telavancin was developed by Theravance and received FDA approval in 2013 [59]. Telavancin is administered intravenously and is associated with adverse effects such as diarrhea, taste disturbance, nausea, vomiting, and discoloration of urine [57].

### 2.6. Dalbavancin (Dalvance)

**Figure 11:**
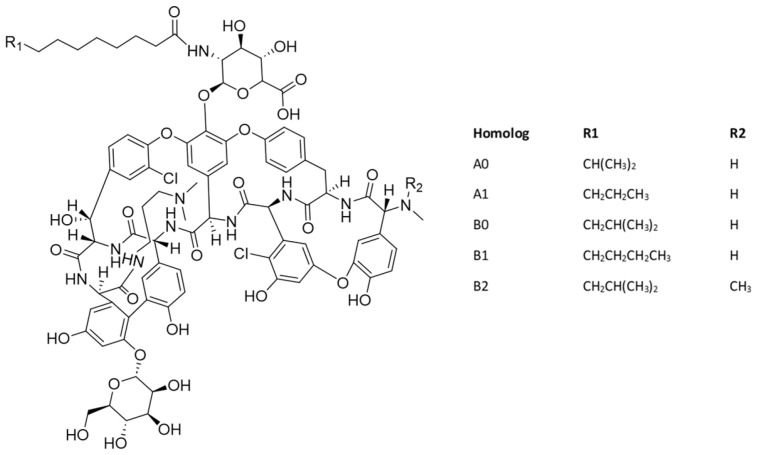
Chemical structure of dalbavancin.

Dalbavancin is a semisynthetic lipoglycopeptide consisting of a mixture of five closely related active homologs (A0, A1, B0, B1, and B2). Among these, component B0 is the major constituent of dalbavancin. The variations lie in the fatty acid side chain of the *N*-acylaminoglucuronic acid moiety (R1) structure and/or the presence of an additional methyl group (R2) on the terminal amino group (Figure 11). Dalbavancin is used in the treatment of acute bacterial skin and skin structure infections (ABSSSIs) caused by designated susceptible strains of Gram-positive microorganisms [60].

Dalbavancin has a mechanism of action similar to vancomycin (Figure 5). It disrupts cell wall synthesis by binding to the D-alanyl-D-alanine terminus of the stem pentapeptide in nascent cell wall peptidoglycan. This interaction prevents cross-linking, ultimately halting the process of cell wall synthesis [60,61].

Dalbavancin was developed by Durata Therapeutics and received FDA approval in 2014 [62]. Dalbavancin is administered intravenously and may be associated with adverse effects such as nausea, headache, and diarrhea [60].

### 2.7. Oritavancin (Kimyrsa)

**Figure 12:**
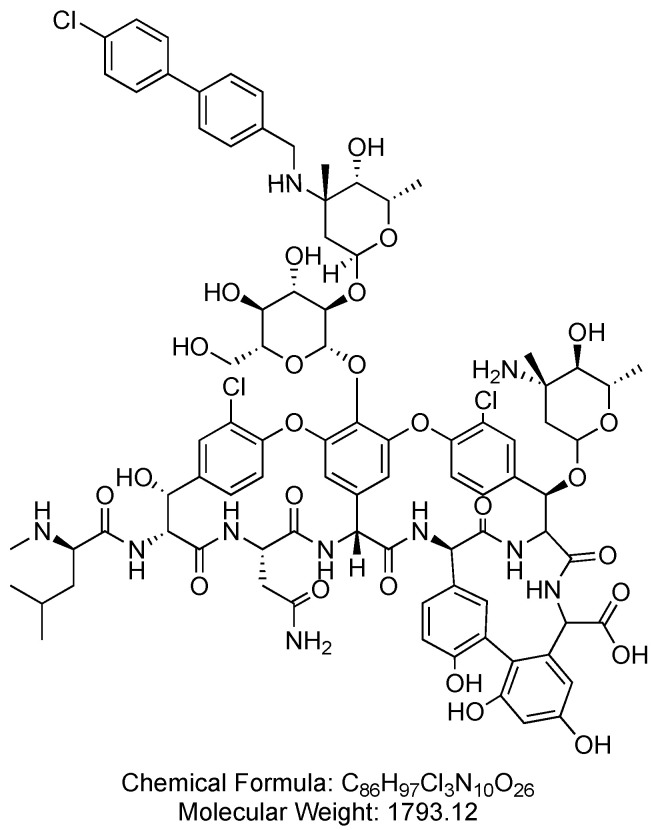
Chemical structure of oritavancin. CID. 16136912.

Oritavancin is a lipoglycopeptide antibacterial drug that distinguishes itself from vancomycin through the incorporation of an aromatic lipophilic side chain and an unsubstituted sugar (Figure 12). Oritavancin is used in the treatment of adult patients with acute bacterial skin and skin structure infections caused or suspected to be caused by susceptible isolates of designated Gram-positive microorganisms [63].

Oritavancin exerts its effects through three mechanisms: (i) inhibition of the transglycosylation (polymerisation) step in cell wall synthesis by binding to the stem D-alanyl-D-alanine peptide of peptidoglycan precursors, (ii) inhibition of the transpeptidation (cross-linking) step in cell wall biosynthesis by binding to the peptide bridging segments of the cell wall, and (iii) disruption of bacterial membrane integrity, resulting in depolarization, permeabilization, and ultimately, cell death [63,64].

Oritavancin was developed by Melinta Therapeutics and obtained FDA approval in 2015 [65]. Oritavancin is administered intravenously and may be associated with side effects such as headache, nausea, vomiting, limb and subcutaneous abscesses, and diarrhea [63].

## 3. Echinocandins Analogs

**Figure 13:**
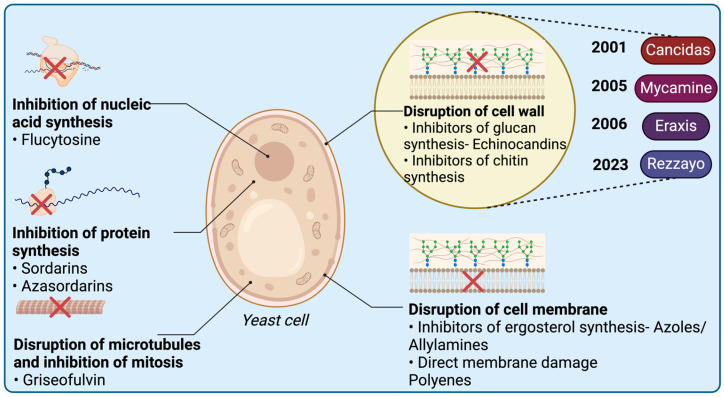
Echinocandins sites of action. Echinocandins inhibit β-(1,3)-D-glucan synthase at the Fks1p subdomain, disrupting fungal cell wall synthesis, leading to osmotic instability and cell death. Created with www.biorender.com (accessed on 19 January 2025).

Echinocandins analogs play a crucial role in targeting and inhibiting *β*-(1,3)-D-glucan synthase, specifically at the Fks1p extracellular subdomain [66]. This subdomain is an integral part responsible for constructing the fungal cell wall [67]. The inability of the organism to synthesize *β*-(1,3)-D-glucan results in osmotic instability and eventual cell death (Figure 13) [68,69,70,71].

These analogs are semisynthetic cyclic lipopeptides exhibiting antifungal activity. They are acylated with different fatty acids attached to the α-amino group of dihydroxyornithine, facilitating the attachment of the drug to the cell membrane of the therapeutic target [72].

This class of drugs traces its roots back to 1992 when caspofungin was initially synthesized from pneumocandin B_0_ and subsequently approved for clinical trials [73]. Since 2001, the FDA has approved four analogs from this class (Table 2).

### 3.1. Caspofungin (Cancidas)

**Figure 14:**
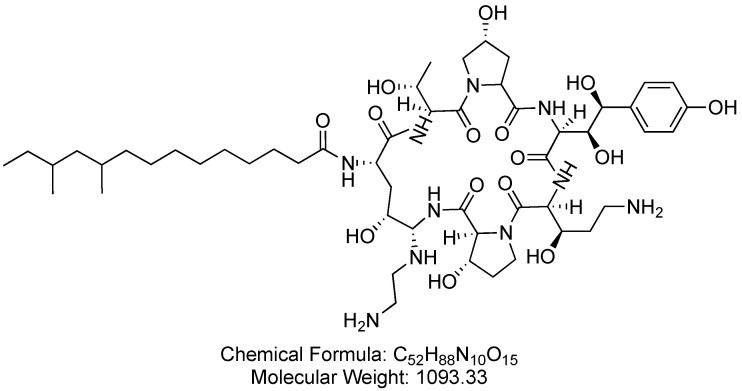
Chemical structure of caspofungin. CID. 16119814.

Caspofungin is a cyclic lipopeptide and an echinocandin antifungal agent that functions as a *β*-1,3-D-glucan synthase inhibitor (Figure 14). Caspofungin is used in the treatment of severe fungal infections, encompassing conditions such as candidemia (fungal infection in the blood), esophageal candidiasis (fungal infection of the esophagus), other candida infections, and aspergillosis (fungal infection in the lungs) [74].

Caspofungin inhibits *β*-1,3-D-glucan synthase, thereby halting the production of *β*-1,3-D-glucan, a crucial component of fungal cell walls. This disruption compromises the structural integrity of the fungal cell walls, ultimately impeding fungal growth and leading to the control of the infection [75].

Caspofungin was developed by Merck Laboratories and became the first within the echinocandin class to receive FDA approval in 2001 [76]. Caspofungin is administered intravenously and may be associated with adverse effects such as chills, fever, phlebitis/thrombophlebitis, tachycardia, nausea, vomiting, rash, abdominal pain, headache, and diarrhea [74].

### 3.2. Micafungin (Mycamine)

**Figure 15:**
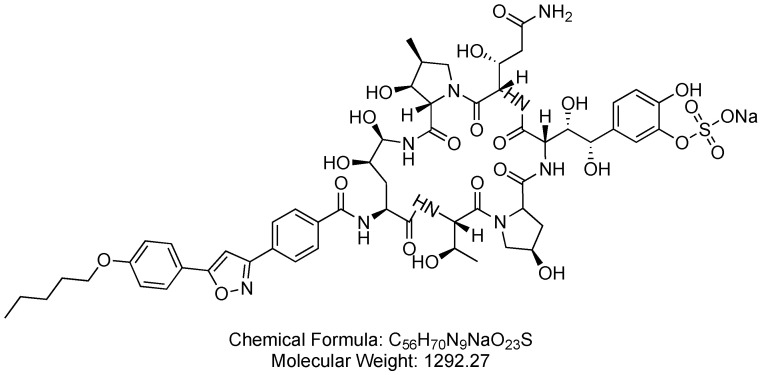
Chemical structure of micafungin. CID. 477468.

Micafungin is a cyclic lipopeptide and an echinocandin antifungal agent that acts as a *β*-1,3-D-glucan synthase inhibitor. Micafungin is utilized to assist the body in overcoming severe fungal infections, including conditions such as candidemia (Figure 15) [77].

Micafungin functions by inhibiting *β*-1,3-D-glucan synthase, disrupting the production of *β*-1,3-D-glucan, a crucial component of fungal cell walls. This disruption compromises the structural integrity of the fungal cell walls, ultimately leading to the control of the fungal infection [77,78].

It was developed by Fujisawa Healthcare and received FDA approval in 2005 [79]. Micafungin is administered intravenously and may be associated with adverse effects such as anxiety, black or tarry stools, bleeding gums, bloating or swelling of the face, arms, hands, lower legs, or feet, cold sweats, coma, and cool, pale skin. Additionally, micafungin can lead to a decreased frequency or amount of urine [77].

### 3.3. Anidulafungin (Eraxis)

**Figure 16:**
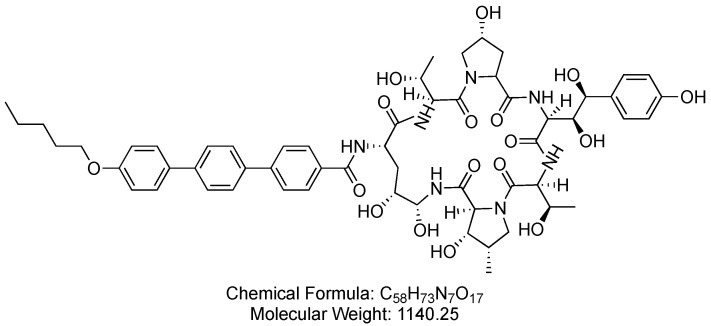
Chemical structure of anidulafungin. CID. 166548.

Anidulafungin is a cyclic lipopeptide and an echinocandin antifungal agent that operates as a *β*-1,3-D-glucan synthase inhibitor (Figure 16). Anidulafungin is used in the treatment of patients with esophageal candidiasis [75].

Anidulafungin inhibits *β*-1,3-D-glucan synthase, disrupting the production of *β*-1,3-D-glucan, which is an essential component of fungal cell walls. This interference compromises the structural integrity of the fungal cell walls, leading to the control of the fungal infection [75].

Anidulafungin was developed by Vicuron Pharmaceuticals and received FDA approval in 2006 [80]. Anidulafungin is administered intravenously, may be associated with adverse effects including black or tarry stools, chills, decreased urine, fever, increased thirst, irregular heartbeat, and lower back or side pain. Additionally, anidulafungin might lead to mood or mental changes [81].

### 3.4. Rezafungin (Rezzayo)

**Figure 17:**
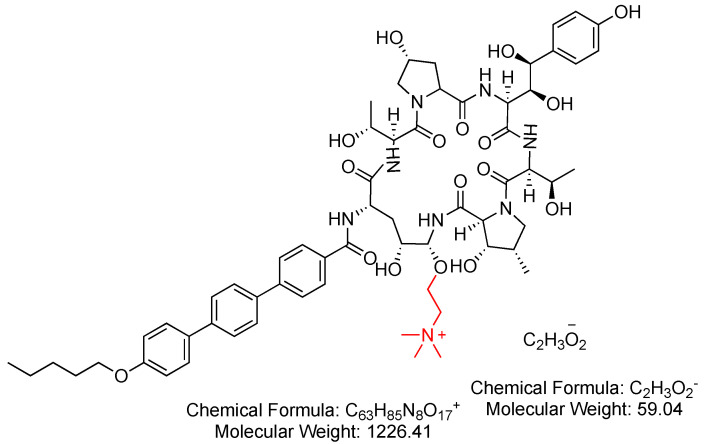
Chemical structure of rezafungin acetate. Difference from anidulafungin is shown in red. CID. 78318119.

Rezafungin is a semisynthetic echinocandin antifungal lipopeptide designed for the treatment of patients aged 18 years or older, particularly those with limited or no alternative options for managing candidemia and invasive candidiasis. Its structure is analogous to that of anidulafungin, with the exception that an OH group in the latter is replaced by a (trimethylammonio)ethoxy moiety in the former (Red) (Figure 17) [82].

Rezafungin functions by inhibiting the 1,3-*β*-D-glucan synthase enzyme complex found in fungal cells. This inhibition results in the prevention of the formation of 1,3-*β*-D-glucan, a crucial component of the fungal cell wall in various fungi, including Candida species. The disruption of 1,3-*β*-D-glucan synthesis contributes to the compromised integrity of the fungal cell wall, ultimately inhibiting fungal growth and aiding in the treatment of infections [82].

Rezafungin was developed by Cidara Therapeutics Inc., and Melinta Therapeutics LLC obtained an exclusive license to commercialize it. Rezafungin received FDA approval in 2023 [83]. Rezafungin is administered intravenously and has demonstrated several adverse effects, including hypokalemia, pyrexia, diarrhea, anemia, vomiting, nausea, hypomagnesemia, abdominal pain, constipation, and hypophosphatemia [82].

## 4. Model Architecture and Methodology

Key experimental databases provide detailed functional activity annotations, such as the Antimicrobial Peptide Database (APD3) (containing 5099 peptides from diverse sources) [83], dbAMP 2.0 (featuring 26,447 AMPs and 2262 antimicrobial proteins from 3044 organisms) [84], LAMP (with 3904 natural and 1643 synthetic AMPs) [85], and AVPdb (including 2683 peptides, 624 of which are modified and tested for antiviral activity). These resources are vital for advancing AMP research and discovery.

Computational methods have emerged as a central focus in bioinformatics, with machine learning-based approaches playing a key role in the identification of AMPs. Advanced techniques such as deep learning and feature extraction have been leveraged to develop numerous models and algorithms, improving the accuracy and efficiency of AMP prediction. Additionally, optimization methods like genetic algorithms, early linguistic models, and QSAR-based models have been employed to generate and optimize AMPs, further advancing their discovery and design [86].

AI methods like CAMPr3, iAMPpred, AmPEPpy, AntiBP2, and CS-AMPPred are widely used for antimicrobial peptide (AMP) discovery. Machine learning (ML) and deep learning (DL) techniques, including random forests, neural networks (NNs), Hidden Markov models, and deep-AMPpred—a two-stage AMP predictor—analyze bacterial phenotypic fingerprints to predict mechanisms of action, estimate antibiotic potency, and assess phenotypic changes. These tools also support the creation of AMP discovery databases, advancing antibiotic research [87,88].

## 5. Conclusions

Peptides have played a crucial role in the pharmaceutical arena for almost seven decades, showcasing their significance. Antimicrobial peptides, for instance, contribute to combating bacterial infections through diverse mechanisms. Glycopeptides have made significant strides in treating MRSA by circumventing PBP mutations. Another noteworthy class, echinocandins, proves effective in treating serious fungal infections. The primary role of peptides as antimicrobial agents is attributed to their mechanism of action, positioning them as a last resort in this field. Peptides predominantly target microbial membranes, thereby circumventing known resistance pathways. Simultaneously, microbes would need to undergo a complete restructuring of their membrane to develop resistance to antimicrobial peptides, an occurrence considered unlikely. Even if resistance were to emerge, the process would likely require an extended period for development.

Despite reluctance in developing new antimicrobial agents, the FDA’s focus on antibiotics reflects the urgency of addressing serious infections. Zevtera, a cephalosporin-based drug approved in 2024, is now approved for treating bloodstream infections, bacterial skin and soft tissue infections, and community-acquired bacterial pneumonia, emphasizing the critical need for innovative solutions [89].

Peptides hold promise through the continuous isolation of new ones exhibiting potent activity against microbes. Moreover, the diverse structures of peptides enable them to exploit various mechanisms, making them versatile agents for addressing both bacterial and fungal infections. In contrast to their small molecule counterparts, peptides can interact with their targets through a larger interface and exert their activity through multiple synergistic mechanisms. Currently, there are around 22 peptide-based therapeutics that are undergoing clinical trials across different phases. These peptides have shown promising results in combating bacterial and fungal infections with a broad spectrum of activity. Moreover, numerous lasso peptides, such as Microcin J25 (MccJ25) and capistruin, demonstrate promising activity against antibiotic-resistant bacteria and hold potential for various applications [90].

Experimental databases, computational tools, and AI-based toolkits are invaluable resources for identifying, engineering, and delivering AMPs capable of overcoming resistance challenges. In addition, the development of a comprehensive computational tool capable of analyzing multiple aspects and activities of AMPs will significantly enhance the discovery of potential AMPs with broad-spectrum capabilities.

## Figures and Tables

**Table 1:** FDA-approved peptide-based antibiotics.

Peptide (Trade Name)	Indication	Therapeutic Target	Route	FDA Approval Year
Common peptide structure				
Gramicidin D (Neocidin)	To treat infected surface wounds, and eye, nose, and throat infections	Bacterial membranes	Lotion or ointment	1955
Glycopeptides				
Vancomycin (Vancocin)	IV: to treat septicemia, infective endocarditis, skin and skin structure infections, bone infections, and lower respiratory tract infections Orally: to treat clostridioides difficile-associated diarrhea and enterocolitis caused by Staphylococcus aureus (including methicillin-resistant strains)	D-alanyl-D-alanine moieties	IV and orally	1958
Lipopeptides				
Colistin (Coly-Mycin M)	To treat infections due to MDR Gram-negative bacteria	Lipopolysaccharide (LPS)	IV	1959
Daptomycin (Cubicin)	For skin and skin structure infections caused by Gram-positive infections	Bacterial membranes	IV	2003
Lipoglycopeptides				
Telavancin (Vibativ)	To treat the following infections in adult patients: complicated skin and skin structure infections	D-alanyl-D-alanine moieties	IV	2013
Dalbavancin (Dalvance)	To treat acute bacterial skin and skin structure infections (ABSSSIs)	D-alanyl-D-alanine moieties	IV	2014
Oritavancin (Kimyrsa)	To treat adult patients with acute bacterial skin and skin structure infections	D-alanyl-D-alanine moieties	IV	2015

IV, intravenous; LPS, lipopolysaccharide; MDR, multidrug-resistant.

**Table 2:** FDA-approved echinocandin peptide analogs.

Peptide (Trade Name)	Indication	Therapeutic Target	Route	FDA Approval Year
Caspofungin (Cancidas)	To treat serious fungal infections	*β*-1,3-D-glucan synthase	IV	2001
Micafungin (Mycamine)	To help the body overcome serious fungus infections, such as candidemia			2005
Anidulafungin (Eraxis)	To treat patients with esophageal candidiasis			2006
Rezafungin (Rezzayo)	To treat patients 18 years of age or older who have limited or no alternative options for the treatment of candidemia and invasive candidiasis			2023

IV, intravenous.

## Data Availability

Data sharing is not applicable.
